# Cholesterol Transporters ABCA1 and ABCG1 Gene Expression in Peripheral Blood Mononuclear Cells in Patients with Metabolic Syndrome

**DOI:** 10.1155/2015/682904

**Published:** 2015-12-15

**Authors:** Zahra Tavoosi, Hemen Moradi-Sardareh, Massoud Saidijam, Reza Yadegarazari, Shiva Borzuei, Alireza Soltanian, Mohammad Taghi Goodarzi

**Affiliations:** ^1^Department of Clinical Biochemistry, Medical School, Hamadan University of Medical Sciences, Hamadan 65178 38736, Iran; ^2^Department of Biochemistry, Medical School, Tehran University of Medical Sciences, Tehran 14176 13151, Iran; ^3^Research Center for Molecular Medicine, Hamadan University of Medical Sciences, Hamadan 65178 38736, Iran; ^4^Department of Genetics and Molecular Medicine, Hamadan University of Medical Sciences, Hamadan 65178 38736, Iran; ^5^Department of Internal Medicine, Medical School, Hamadan University of Medical Sciences, Hamadan 65178 38736, Iran; ^6^Department of Biostatistics & Epidemiology, School of Public Health, Hamadan University of Medical Sciences, Hamadan 65178 38736, Iran

## Abstract

ABCA1 and ABCG1 genes encode the cholesterol transporter proteins that play a key role in cholesterol and phospholipids homeostasis. This study was aimed at evaluating and comparing ABCA1 and ABCG1 genes expression in metabolic syndrome patients and healthy individuals. This case-control study was performed on 36 patients with metabolic syndrome and the same number of healthy individuals in Hamadan (west of Iran) during 2013-2014. Total RNA was extracted from mononuclear cells and purified using RNeasy Mini Kit column. The expression of ABCA1 and ABCG1 genes was performed by qRT-PCR. Lipid profile and fasting blood glucose were measured using colorimetric procedures. ABCG1 expression in metabolic syndrome patients was significantly lower (about 75%) compared to that of control group, while for ABCA1 expression, there was no significant difference between the two studied groups. Comparison of other parameters such as HDL-C, FBS, BMI, waist circumference, and systolic and diastolic blood pressure between metabolic syndrome patients and healthy individuals showed significant differences (*P* < 0.05). Decrease in ABCG1 expression in metabolic syndrome patients compared to healthy individuals suggests that hyperglycemia, related metabolites, and hyperlipidemia over the transporter capacity resulted in decreased expression of ABCG1. Absence of a significant change in ABCA1 gene expression between two groups can indicate a different regulation mechanism for ABCA1 expression.

## 1. Introduction

The metabolic syndrome (MetS) is defined as a set of interrelated risk factors of diabetes and cardiovascular diseases (CVD) [1]. There are some criteria for clinical diagnosis of metabolic syndrome that include elevated waist circumference (≥102 cm in men and ≥88 cm in women), elevated triglycerides (≥150 mg/dL or 1.7 mmol/L), reduced high density lipoprotein-cholesterol (HDL-C <40 mg/dL or 1.03 mmol/L in men and <50 mg/dL or 1.3 mmol/L in women), and elevated blood pressure (≥130 mm Hg systolic blood pressure or ≥85 mmHg diastolic blood pressure) [2]. The MetS can be diagnosed by observation of three of these criteria. In the past several years, the prevalence of MetS increased worldwide [3, 4]; however, in the United States of America, it has declined from 25.5% in 1999/2000 to 22.9% in 2009/2010 [5]. Weiss and colleagues reported that obesity is directly associated with increased prevalence of MetS [6]. There is a reverse association between the CVD and plasma HDL-C level, one of the metabolic syndrome criteria [7, 8]. High density lipoprotein, a subfraction of circulatory lipoproteins, plays an important role in cholesterol transport from peripheral tissue to liver cells [9]. HDL is rich in Apo A-I and Apo A-II proteins, and more than two-thirds of its content is Apo A-I [10, 11].

The ABC transmembrane transporters have an important role in cholesterol uptake from macrophage to HDL and decrease the formation of foam cells [10]. ABCA1 composed of 2261 amino acids [12] presents in most tissues. In recent years, it has been shown that ABCA1 plays an essential role in protecting from cardiovascular disease [1]. HDL synthesis directly depends on ABCA1 activity in liver cells; in other words, it has a key function in arterial cells protection against foam cells through increasing plasma HDL. Arterial macrophages ABCA1 activity has shown a reverse association with foam cell formation, as with the increase in its activity the formation of foam cell will reduce [11].

The reduced or impaired ABCA1 activity could cause some diseases such as type 2 diabetes [13], Tangier disease [14, 15], and premature CVD [16, 17].

ABCG1 gene is located on chromosome 21q22.3 [18]. Both ABCA1 and ABCG1 lead to reduction of tissues cholesterol by its efflux to HDL, but ABCG1 transports tissue cholesterol to HDL_2_ and HDL_3_ and ABCA1 transports it to lipid-free Apo A-I [19, 20]. Macrophages are the most important tissue of ABCA1 and ABCG1 action [21]. In many studies, the effects of upregulation and downregulation of these genes were examined.

Reduced cholesterol efflux is the consequence of lacking ABCA1 expression in vitro [22, 23]; it can lead to increase in atherosclerosis [23], but upregulation of ABCA1 results in reduction of atherosclerosis [24]. ABCG1 downregulation also has the same effects on cholesterol efflux but there are controversial results about the impact of ABCG1 downregulation on atherosclerosis [25–27].

In MetS, the patterns of expression of some genes change and can lead to obesity, diabetes, and hypertension. In the present study, we aimed to evaluate the ABCA1 and ABCG1 genes expression in patients with MetS, since these genes are involved in the transporters synthesis which have a crucial role in cholesterol transport.

## 2. Materials and Methods

### 2.1. Subjects

This case-control study was carried out on patients that were referred to an endocrinology ward in a hospital in Hamadan (west of Iran) during 2013-2014. Thirty-six patients with metabolic syndrome were selected. Also, 36 age- and sex-matched healthy individuals were selected as control group. None of the healthy individuals had the criteria of metabolic syndrome.

The inclusion criterion in metabolic syndrome group was having three out of five of the abovementioned characteristics. The patients with history of consumption of antilipid, contraceptive, and diuretic drugs were excluded from the study. The pregnant patients and patients with diabetes, inflammation, and infection were excluded too.

### 2.2. Blood Sampling

A 2.5 mL blood sample of each subject was added to an EDTA-containing tube and was kept at 4°C for RNA extraction (no more than two hours later).

### 2.3. Extraction of Peripheral Blood Mononuclear Cells (PBMCs)

Lymphodex (Germany) and Henx (Iran) solutions were used for isolation of PBMC. About 2 mL of Henx solution was added to equal volume of blood and blended completely; then it was poured onto 3 mL Lymphodex solution carefully and slowly. Subsequently, the mixture was placed on Lymphodex and was centrifuged at 1000 g for 20 minutes. The intermediate white layer between the plasma and Lymphodex was isolated as mononuclear cells (MCs); then the Henx buffer was added on MC, mixed completely, and centrifuged. Finally, the supernatant was discarded and the process was repeated once more.

### 2.4. RNA Extraction and cDNA Synthesis

The net extraction of RNA was performed using the Gene JET RNA Purification kit according to the manufacturer protocol. The quality and quantity of the purified RNA were analyzed using NanoSpectrophotometer (Epoch, BioTek, USA); then the integrity of each RNA sample was examined by using 1% agarose gel, 1x TBE.

The RNA was converted to cDNA using RevertAid First Strand cDNA Synthesis Kit (K1622) through following the protocol: step 1: primer annealing at 25°C for 5 min; step 2: cDNA synthesis at 42°C for 60 min; step 3: heat inactivation at 70°C for 5 min. The products were stored at −80°C for next steps.

### 2.5. Primer Design

The specific primers for each gene were designed by Allele ID software (version 7.6). In order to increase the specificity of real-time PCR reaction and reduce the false positive results, one of each pair primer was designed to be attached to Exon-Exon junction area. The thorough criteria of the used primers for each gene are presented in Table 1. The 18S rRNA housekeeping gene was used as an internal control.

The relative expression of the genes was calculated through measuring the threshold cycle (CT) value for each sample using C1000 Thermocycler and CFX96 real-time system (Bio-Rad, USA) and SYBR Premix Ex Taq 2 Kit (TakaRa No. RR820L). The average of CT in triplicate assay of each sample was determined as CT value. The ingredient amounts of qRT-PCR reaction included 10 *μ*L SYBR green, 7 *μ*L deionized water, and 1 *μ*L of each of the forward primer, reverse primer, and template. qRT-PCR was performed in these conditions: initial activation at 95°C for 30 sec and then 40 cycles of the following steps repeated: denaturation at 95°C for 5 sec, annealing at optimized annealing temperature for proper duration of each gene, and extension at 72°C for 30 sec, and at the end, the data were acquired by increasing temperature from 72°C to 95°C for 0.5°C/0.05 sec. Then, the PCR products were electrophoresed (on 1% agarose gel, 1x TBE) to verify the specificity of amplicons.

### 2.6. Evaluation of the Serum Biochemical Factors

A two-milliliter blood sample was collected from each subject and centrifuged at 3000 rpm for 10 min for serum separation. Total cholesterol (TC), LDL-C, TG, FBS, and HDL-C were examined using Pars Azmun (Iran) kit by Hitachi 911 (Germany).

### 2.7. Analysis and Interpretation of Results

2^−ΔΔCT^ formula was used for the analysis of the relative gene expression of metabolic syndrome and control groups. The efficiency of real-time PCR was calculated for each gene by using 10^−2^ dilution; subsequently, the obtained number was placed at the following computational formula for measuring the fold changes of gene expression [28]:

(1) ΔCT=CTtarget−CTreference,ΔΔCT=ΔCTcase−ΔCTcontrol,Fold  Change=2−ΔΔCT.

SPSS V.16 software was used for statistical analysis with 95% confidence intervals. Normal distribution of the variables was checked by Kolmogorov-Smirnov method and then the values were compared between two groups via independent samples *t*-test.

## 3. Result

Demographic characteristics and biochemical factors of the studied groups are presented in Table 2. The male/female ratio was 1/3 in each group (12 M and 24 F). As shown in Table 2, the differences in all examined parameters were statistically significant between two groups (*P* < 0.05) with the exception of age and LDL-C. The metabolic syndrome group had higher BMI, LDL-C, TC, TG, FBS, diastolic/diastolic blood pressure, and circumference waist compared to healthy individuals, while HDL-C was significantly lower in metabolic syndrome.

### 3.1. Results of qRT-PCR

**Figure 1:**
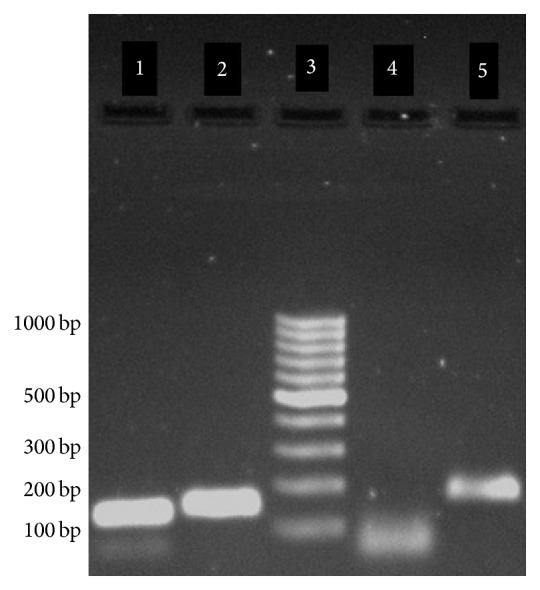
Agarose gel electrophoreses of RT-PCR products: lane 1, ABCG1 products with 135 bp; lane 2, 18S RNA product with 151 bp; lane 3, molecular weight standards with 100–1000 bp; lane 4, negative control with <100 bp; lane 5, ABCA1 products with 180 bp.

After completion of PCR reaction, the results were verified by analysis of the reaction curve, melting curve of products, and electrophoresis of the PCR products. The electrophoresis was run on 1% agarose using a 100 bp DNA ladder. The results are shown in Figure 1.

### 3.2. Evaluation of Gene Expression in PBMC

The expression levels of the genes were measured in the peripheral blood mononuclear cells and compared between two groups by calculation of ΔCT for each gene. There was no difference in ABCA1 expression between two groups but ABCG1 expression was significantly lower in MetS group (*P* = 0.02). The detailed results are shown in Table 3.

Also, the calculation of fold change in ABCG1 expression (2^−ΔΔCT^) indicated 3.1-fold lower expression in MetS group.

## 4. Discussion

ABCA1 and ABCG1 have a crucial role in cholesterol reverse transport from peripheral tissues such as macrophages to liver [29]. However, ABCA1 and ABCG1 genes expression has been studied in some diseases; we did not find any report on this subject in metabolic syndrome. In this study, we examined the expression of these genes in metabolic syndrome subjects and healthy individuals.

While there was no significant difference in ABCA1 gene expression between two studied groups, we found significantly lower expression (about 75%) of ABCG1 in subjects with metabolic syndrome compared to control group. Singaraja et al. showed that the reduced ABCA1 in macrophages and kidneys is associated with increased cholesterol content [30]. They concluded that impaired ABCA1-mediated cholesterol export could contribute to the increased atherosclerosis and nephropathy associated with diabetes [30]. There are some reports trying to address the possible mechanisms that regulate the expression of these genes. Recently, it has been shown that polymorphisms in ABCA1 and ABCC8 may be associated with metabolic syndrome [31]. There is evidence showing that disruption in ABCA1 function, which can be the result of mutation in this gene, can lead to familial hypoalphalipoproteinemia that is characterized by low HDL and increased deposition in cholesteryl esters in several tissues and cells [32]. Also, it has been shown that overexpression of ABCA1 gene can confer protection against atherosclerosis [33]. These data are consistent with our findings and support this idea that lower expression of ABCA1 gene may contribute to metabolic syndrome complications.

Haghpassand et al. demonstrated that ABCA1 gene expression in PBMCs has direct relationship with the load of cholesterol, and overexpression of ABCA1 leads to transfer of the excess cholesterol to the apoproteins [22]. Wang et al. studied the reverse cholesterol transport in ABCA1 and ABCG1 knockdown mice and concluded that when both ABCA1 and ABCG1 were knocked down, there is greater reverse cholesterol transport compared to ABCG1 knocked down [29].

Since Mauerer et al. pointed out that the high level of glucose (hyperglycemia) is involved in reduced ABCA1 and ABCG1 expression in vitro [34], it seems logical to expect similar changes in MetS. Promoters of ABCA1 and ABCG1 have receptor site for LXR and RXR; when oxycholesterol and retinoic acid join these factors, expression of ABCA1 and ABCG1 is increased. Also, cAMP and NF*κ*B are transcriptional factors for ABCA1 and ABCG1, respectively.

The obtained results indicated a significant decrease in ABCG1 expression in metabolic syndrome patients compared with healthy individuals. These results suggest that hyperglycemia, related metabolites, and hyperlipidemia over the transporter capacity may lead to decreased expression of ABCG1. Because no significant change was observed in ABCA1 gene expression, it can be due to a different regulation mechanism. Since structures of these genes are different and they are regulated by diverse transcriptional factors, our results can be justified [35, 36]. Nevertheless, the limited number of the samples in this study is another factor for the absence of changes in ABCA1 expression.

The other point is that we examined the expression of these genes in PBMC of the studied subjects; it is important to notice that the changes in the expression of these genes in monocytes may be different from that in the other key tissues such as liver and intestine, which are the major sites of HDL synthesis. Furthermore, the changes in mRNA levels do not necessarily imply changes in the related protein.

## Figures and Tables

**Table 1:** Sequence, *T*_*m*_, and product length of the used primers in this study.

Gene name	Primer sequence	Product length (bp)	*T*_*m*_ (°C)
ABCA1	F: 5′-TGCAAGGCTACCAGTTACATT-3′	180	79
	R: 5′-TTAGTGTTCTCAGGATTGGCT-3′		

ABCG1	F: 5′-AAGGTGTCCTGCTACATCAT-3′	135	81.5
	R: 5′-CAGTATCTCCTTGACCATTTC-3′		

18S rRNA	F: 5′-dGTAACCCGTTGAACCCCATT-3′	151	64.5
	R: 5′-dCCATCCAATCGGTAGTAGCG-3′		

**Table 2:** Basic characteristic of the studied groups.

Factors	Control	Metabolic syndrome	*P* value
	(mean ± SD)	(mean ± SD)	
Age (year)	48.5 ± .25	50.0 ± 2.02	0.222
Waist circumference (cm)	95 ± 1.8	106 ± 3	0.001^*∗*^
BMI (kg/m^2^)	25.5 ± 0.8	30.0 ± 0.9	0.002^*∗*^
Systolic blood pressure (mmHg)	120.2 ± 1.9	130.5 ± 1.6	0.003^*∗*^
Diastolic blood pressure (mmHg)	80.5 ± 2.1	85.4 ± 1.1	0.006^*∗*^
FBS (mg/dL)	91.5 ± 1.62	108 ± 3.63	0.001^*∗*^
TG (mg/dL)	141 ± 10.5	187.5 ± 16.0	0.015^*∗*^
HDL-C (mg/dL)	50.5 ± 1.35	42.63 ± 1.50	0.001^*∗*^
Total cholesterol (mg/dL)	189 ± 8.1	213 ± 8.0	0.039^*∗*^
LDL-C (mg/dL)	118 ± 8	128.5 ± 6	0.096

BMI: Body Mass Index; FBS: Fast Blood Sugar; TG: triglyceride, HDL-C: high density lipoprotein-cholesterol; LDL-C: low density lipoprotein-cholesterol; ^*∗*^significant difference.

**Table 3:** Comparison of ΔCT of ABCA1 and ABCG1 genes between two studied groups.

Gene	Groups		*P* value
	Control	Metabolic syndrome	
ABCA1	12.60 ± 0.60	12.19 ± 0.26	0.539
ABCG1	14.63 ± 0.60	12.98 ± 0.33	0.020^*∗*^

^*∗*^Significant.
